# Assessing social support networks in cancer survivors and their care partners: concordance between expected and received support

**DOI:** 10.1007/s11764-025-01880-8

**Published:** 2025-08-19

**Authors:** Kristin G. Cloyes, Jia-Wen Guo, Kelly Jean Mansfield, Sarah Nowak, Maija Reblin

**Affiliations:** 1 School of Nursing, Oregon Health & Science University, Portland, OR USA; 2 College of Nursing, University of Utah, Salt Lake City, UT USA; 3 Larner College of Medicine, University of Vermont, Burlington, VT USA

**Keywords:** Cancer survivor, Caregiver, Social network, Social support

## Abstract

**Purpose:**

Cancer survivors and care partners rely on their personal social networks for emotional, informational, instrumental, and belonging support. We examined concordance between expected and received support by assessing how participants’ initial reports of which network members would provide specific types of support aligned with the support they reported receiving over time.

**Methods:**

At baseline, 32 survivors diagnosed within the past 5 years and their care partners (*N* = 64) identified 7–20 network members and the types of support each routinely provided. Participants completed repeated interaction surveys over 3 months, describing interactions with network members and the type of support received. Concordance was calculated by comparing baseline expectations to subsequent reports, and associations with participant demographics, network characteristics, and interaction patterns were examined.

**Results:**

Concordance across all participants and support types was 38%. Individually, average concordance was 57%, with wide variability. Concordance was highest for emotional support and lower for informational and practical support. Higher concordance was associated with more frequent interactions and fewer unhelpful or absent support experiences. No significant associations were found with participant demographics or structural network features.

**Conclusions:**

Survivors and care partners may be challenged to anticipate how support from their personal networks will unfold over time. Concordance varied by support type and was more closely related to quality and frequency of support interactions than structural network characteristics.

**Implications for Cancer Survivors:**

Interventions that help survivors and care partners assess their support resources and variation over time may improve alignment between support needs and experiences, enhancing well-being.

## Introduction

Social support is a social determinant of health [1] and a vital component of better health and wellbeing outcomes for both cancer survivors and their primary care partners, those family members, friends, and other people in their lives who provide support along the survivorship trajectory [2]. Social support is broadly defined as the assistance, care, attention, and encouragement people receive through their relationships, social connections, and interactions with other people. Prior research spanning decades [3] has led to the identification and definition of different types of social support that are commonly defined in social support and social network research, including those defined by Cohen et al. (1985) in their work on the often-cited stress-buffering model: instrumental (e.g., practical and tangible material forms of support), informational (e.g., sharing advice and knowledge helpful to appraising and coping with problems), emotional (e.g., trust, empathy, and feeling that others care for you), and belonging or esteem support (e.g., feeling included and like a valued member of a group that shares common interests) [4]. Later social support research identified subtypes of support falling within these categories (e.g., appraisal) [3, 5–8]. Commonly used measures of functional social support, such as the Duke-UNC Functional Social Support Questionnaire (FSSQ) [9] and the Multidimensional Scale of Perceived Social Support (MSPSS) [10], continue to measure functional support with subscales based on instrumental, informational, and emotional forms of support.

Social support has been characterized in three different but related ways [11]. Structural social support research focuses on quantifiable characteristics of the support network such as how many people are in one’s network, number of connections among members, qualities of relationships between members (e.g., strength of connections), availability of members, and the ratio of family to non-family members [12]. Functional social support refers to how networks provide cancer survivors and care partners with social support [13].

Personal social support networks are the dynamic and unique set, or network, of social relationships each person has with other people in their lives through which they access and engage different kinds of support. While each cancer survivor and care partner are at the “center” of their own individual set of connections and relationships, the people within an individual’s social network may be connected to and provide support for others within that network [8]. For example, family members and close or long-term friends within one’s social network are often connected with each other, while acquaintances, coworkers, and other more peripheral relationships, particularly those characterized by less frequent and more occasional interaction, may be connected to the survivor or care partner but not to other people in their network.

Social support provided via connections within one’s social support network is vital for the health and well-being of both cancer survivors and their care partners. Engagement with social network members can reduce stress, increase coping, and have positive effects on outlook, quality of life, and health-related self-management [14–17]. Moreover, social support research has demonstrated that perceived support can often drive health outcomes more than received or enacted support [3]. This may be because support from social relationships is often actively sought and engaged when individuals face adversity, such as challenges in managing a chronic or serious illness; enacted support may demonstrate the responsiveness of one’s social network to assist when needed [4]. Similarly, support matching—or offering the appropriate type of support that matches the desired or needed help—can be critical to ensuring that support is effective [5], including in cancer and caring contexts [6, 7]. Throughout daily life, individuals may form ideas about what kinds of support will be available from different social network members [8]. These ideas, however, may not align with the actual dynamics of how social relationships work in daily life over time; this often becomes a challenge because these assumptions may be tested in times of stress [9]. Life-altering events like cancer can intensify the need for social support to help cope with the implications of diagnosis, symptoms, treatment, and emotional and social impact, including taking on new roles for both survivors and their care partners [10, 11]. However, cultural and interpersonal factors can shape whether support needs are met. For example, it can be difficult for survivors or their care partners to ask for specific forms of help, and family, friends, neighbors, or other social contacts may be unsure about what is needed or how they can best provide support that will be welcome and meaningful to survivors and their care partners [12]. When a patient or care partner has functional limitations, such as illness or fatigue, or logistical constraints, such as time-consuming treatments, this can also present challenges; they may be unable to explain their needs to people who could offer support or actively engage or maintain social connections with potentially helpful connections within their social support network [13, 14].

Additionally, cancer survivors’ ability to accurately assess or predict the availability of different kinds of social support, from different sources within their networks, may not always align with the dynamic nature of social relationships. That is, their expectations may not be concordant with their experiences or their needs. Not all our network members are equipped to provide every type of support we may need, nor are all types of support desired from each person in our social support network. [15, 16] For example, someone living far away may be unable to provide instrumental (or practical) support, such as rides to and from doctor appointments, while informational or emotional support that strikes the wrong tone (e.g., making assumptions about experiences of the survivor or care partner and being overly optimistic or pessimistic) may be seen as unhelpful.

Further, the types of support offered by specific people in our support network can also change in response to significant events and the vacillations of daily life. A close friend may be a reliable source of support most of the time, but if they become ill or are facing their own challenges, they may be temporarily unable to offer any support. A visiting friend-of-a-friend may be available and willing to make dinner, eliminating the need for a close family member to shop and cook. Expectations for support can also be informed by previous behavior; if a friend has a poor or unpredictable history of providing support, they may not be approached for future support.

Moreover, when support comes from people with whom survivors or care partners share more extensive personal history and closer ties, such as family members, these relationships can be laden with expectations, memories, and stress/support ambivalence that significantly complicates both perceptions of and engagement with available resources [17, 18]. Retrospective, single-timepoint measures may not capture these real-world dynamics and complexities. They also do not measure alignment between survivors’ or care partners’ expectations of support and from whom they receive support.

### Study purpose

To better understand cancer survivors’ and care partners’ social support resources, we examined the concordance between participants’ assessments of their social support networks at baseline, including who in their network typically provided different types of support, and their reports of actual interactions with these specific network members and the types of support these network members provided them over a 3-month period. Specifically, we (a) assessed structural characteristics of their social support networks, (b) prospectively examined functional characteristics of their social support interactions with network members, (c) calculated the concordance between participants’ baseline expectations and their reports of support interactions with network members, and (d) explored associations of concordance scores with participants’ demographics and social support network characteristics (e.g., network size, number of interactions between participants and members, and types of social support perceived/given in each interaction).

## Materials and methods

This observational prospective cohort study combined social network analysis and daily surveys on characteristics of interactions with network members, a form of experience sampling for prospective research [18] that allowed us to compare structural information about participants’ personal social support networks with daily patterns of participant interactions with network members over time. The University of Utah Institutional Review Board reviewed and approved all study activities.

### Recruitment

We recruited both cancer survivors and the person they identified as their primary informal caregiver (“care partner”) during their most recent cancer experience. Cancer survivors were recruited first, via study flyers and emails that were distributed via social worker-led support groups, clinic email lists, and the social media channels (e.g., Facebook and Instagram) of Huntsman Cancer Institute, an NCI-designated cancer center in the U.S., and ResearchMatch, a U.S.-based national registry of health-related research volunteers. Eligibility for survivors included being ≥ 18 years of age, diagnosed with any cancer type within the past 5 years, not currently living in a residential care setting, able to read and speak English, and able to identify an eligible care partner who was their primary cancer-related support person; this identified care partner also consented to participate. Care partners could have any relationship with the survivor, live with the survivor or not, and share the same eligibility criteria except for a cancer diagnosis.

Interested participants individually contacted the research team for an initial phone or video conference screening to ensure that both study partners met inclusion criteria, and both wanted to participate [19]. Those who qualified were sent a link to an online consent form and baseline demographic and social network surveys. Recruitment and enrollment continued until we reached our target sample size of n-32 survivor-care partner dyads. Because of the exploratory nature of this study, and the amount of data generated via our prospective approach to collecting participant-network interaction data (e.g., ≥ 600 network members identified), this sample size was determined to be adequate for aims to assess support network characteristics and compare participants’ baseline expectations to reported interactions over the study period [20]. Once survivors and care partners completed screening and consent, each was sent a separate link to the baseline study surveys. Participants received $150 compensation for completing the study: $50 for completing baseline measures and the first week of daily interaction surveys, and $50 each for completing weeks 2 and 3 of daily interaction surveys.

### Data collection

We used REDCap [20, 21] to collect baseline and daily interaction survey data; participants were sent a link to REDCap baseline surveys and then opted to receive the REDCap daily interaction surveys via email or text message. We used EgoWeb [22], an open-source social network assessment program, in a participant-facing mode where participants completed baseline social network assessments independently.

#### Baseline and social network measures

Participants completed a demographic survey and assessed the structural characteristics of their personal support network at baseline. Each participant was asked to identify between seven and 20 people in their social support network (*people in your life who provide you with personal support*). For each person listed, participants were asked to provide the following information: age, race, ethnicity, gender, sexual orientation, relationship (relative or non-relative), and length of time known. We provided definitions for different types of support based on established categories in the social support literature, drawing from validated instruments such as the FSSQ [9] and other commonly used measures of functional support (instrumental, informational, emotional, and belonging). We asked participants the type/s of support each person typically provided (participants could mark multiple types of support for each person) and to rate the availability of that network member to offer support.

#### Daily interaction surveys

The social support network data collected at baseline was then used to personalize and populate daily surveys asking participants to report on their social support interactions with their baseline-identified social support network members. Participants were sent links to the daily interaction survey every day for seven days after completing the baseline measures (week one). This survey asked them to select specific baseline-identified social support network members they interacted with that day and report on characteristics of those interactions, including modality (e.g., in-person, phone, email, and text), whether they perceived the interaction as providing social support, and the type/s of social support provided. During weeks 5 and 9, participants were sent the daily interaction survey again for seven days for a total of 21 days of daily interaction data. Each day, participants reported which network members they interacted with and the type/s of support the person provided during the interaction and then completed a Likert item rating the interaction as either helpful or not helpful or if support was not the purpose of the interaction.

### Data analysis

#### Social support network and interaction characteristics

We used descriptive statistics to summarize participant demographics, social support network characteristics, and interaction data. We calculated four social support network variables for each participant [21]: (1) network size, a count of the number of people participants identified as their social support network members minus the participant; (2) network density, a measure of how connected members are to each other, calculated by dividing the number of actual connections of members to other members by the number of possible connections; (3) modularity, a measure of how much the social support network divides into subgroups where members are more strongly connected with other members in their subgroup than to members outside it, calculated by comparing the density of within-subgroup connections to what would be expected if connections were randomly distributed across the network; and (4) the proportion of family relationships (e.g., spouse, parent, sibling, and child) vs. non-family relationships (friend, neighbor, and co-worker) represented among network members [12]. R version 4.2.3 was used to generate social network and interaction measures, concordance categories for each network member, and concordance scores. Since several of our network and interaction variables were not normally distributed, we reported median values for descriptive summaries. Mann–Whitney *U* tests were used to compare survivors and care partner groups by support network and interaction characteristics and concordance scores, with a significance level set at 0.05.

#### Calculation of concordance of expected and reported support

Our goal was to operationalize a comparison of perceived and received support by combining retrospective and prospective data. We defined *concordant support* as the proportion of social support network members listed by participants at baseline as a source of a specific type of support (informational, instrumental, emotional, belonging) who provided that specific support type at least once during daily interactions over the 3 months of reporting (i.e., support type expected/support type reported). *Concordant nonsupport* was the proportion not identified as a source of support that did not provide that support type (not expected/not reported). We also defined mismatches between expectations and reported outcomes: *missed opportunities* were the proportion listed as a source that did not provide that support type (expected/not reported); *unexpected support* was the proportion not listed as a source that did provide that support type (not expected/reported).

We calculated the *overall concordance of expected vs. reported support* by counting the social support network members identified by participants at baseline who were also listed in the interaction surveys as providing each specific type of support during the study period. This provided the percentage of network members in each concordance category by support type, and the proportion of interactions that matched participants’ baseline expectations. *Concordance scores* were generated for each participant as the sum of the proportion of network members in the concordant support and concordant nonsupport categories across the four support types. For both measures, possible scores ranged from 0 to 4, and higher scores represented greater accuracy. Our sample’s concordance scores were normally distributed, and we compared survivor and care partner groups using an independent samples *t*-test.

#### Associations of concordance with demographic, network, and interaction characteristics

Finally, we aimed to investigate whether concordance scores were correlated with specific demographic, network, and interaction measures. Specific demographic variables were selected based on previous research suggesting that age, income, relationship status, and network size and structural characteristics (density, modularity, and access to support as measured by frequency or interaction) are associated with perceived and received social support [22, 23]. Spearman’s rho tests were used to calculate correlation and confidence intervals; *P* values were set at 0.05 significance.

## Results

### Participant demographics

Table 1 presents participant demographic characteristics. We recruited *N* = 32 survivor and care partner dyads (*N* = 64 participants). Most were White (*n* = 61, 95.31%), not Hispanic (*n* = 57, 86.06%), and women (*n* = 39, 60.94%), and the average age was 43.28 (SD, 17.18; range, 19–76). Fifty-six percent (*n* = 36) of study dyads were spouses/partners, followed by parent–child dyads (*n* = 20, 31.25%).

**Table 1:** Participant demographics

	**All (*N* = 64)**	**Survivor (*n* = 32)**	**Care partner (*N* = 32)**
	Yrs (SD)	Yrs (SD)	Yrs (26)
Age	43.28 (17.18)	41.34 (18.23)	45.33 (16.12)
Study partner relationship	***N*** (%)	***n*** (%)	***n*** (%)
Spouse/partner	36 (56.25)	18 (56.25)	18 (56.25)
Parent	10 (15.63)	6 (18.75)	4 (12.50)
Sibling	2 (3.13)	1 (3.13)	1 (3.13)
Child	10 (15.63)	4 (12.50)	6 (18.75)
Friend	6 (9.38)	3 (9.38)	3 (9.38)
Gender			
Male	24 (37.50)	8 (25.00)	16 (50.00)
Female	39 (60.94)	23 (71.88)	16 (50.00)
Non-Binary	1 (1.56)	1 (3.13)	–
Sexual orientation			
Heterosexual	62 (96.88)	30 (93.75)	32 (100)
Lesbian/gay	–	–	–
Bisexual	2 (3.13)	2 (6.25)	–
Race			
White	61 (95.31)	30 (93.75)	31 (96.88)
Asian	1 (1.56)	–	1 (3.13)
Multiple Races	2 (3.13)	2 (6.25)	–
Ethnicity			
Hispanic/Latinx	6 (9.38)	5 (15.63)	1 (3.13)
Not Hispanic/Latinx	57 (89.06)	26 (81.25)	31 (96.88)
Prefer not to say	1 (1.56)	1 (3.13)	–
Education			
High school	3 (4.69)	1 (3.13)	2 (6.25)
Some college or vocational school	20 (31.25)	11 (34.38)	9 (28.13)
College graduate	22 (34.38)	10 (31.25)	12 (37.50)
Some graduates or professionals Schooling	5 (7.81)	3 (9.38)	2 (6.25)
Graduate or professional degree	14 (21.88)	7 (21.88)	7 (21.88)
*Employment			
Full time	22 (34.38)	8 (25.00)	14 (42.75)
Part-time	10 (15.63)	3 (9.38)	4 (12.50)
Not working for pay	12 (18.75)	7 (21.88)	4 (12.50)
Medical or other leave	5 (7.81)	3 (9.38)	–
Retired	9 (14.06)	5 (15.63)	3 (9.38)
Student	7 (10.94)	3 (9.38)	1 (3.13)
Self-employed	5 (7.81)	1 (3.13)	4 (12.50)
Household income			
Less than $9999	2 (3.13)	2 (6.25)	–
$10,000-$24,999	4 (6.25)	1 (3.13)	3 (9.38)
$25,000-$39,999	9 (14.06)	5 (15.63)	4 (12.50)
$40,000-$49,999	5 (7.81)	2 (6.25)	3 (9.38)
$50,000-$74,999	10 (15.63)	6 (18.75)	4 (12.50)
$75,000-$99,999	6 (9.38)	3 (9.38)	3 (9.38)
> $100,000	21 (32.81)	8 (25.00)	13 (40.63)
Prefer not to say	1 (1.56)	1 (3.13)	–
Do not know	3 (4.69)	2 (6.25)	1 (3.13)

*Participants could select all applicable categories

### Social support network characteristics

Both social network and interaction characteristics are presented in Table 2. The median network size (number of members not including the cancer survivor or care partner) was 9.0 members (SD = 1.99, range 3–13). Survivor and care partner groups did not significantly differ on any network variables, but survivors’ support networks were slightly larger (*U* = 458.00, *z* =  − 0.725, *P* = 0.468). There were also no differences in network density (*U* = 546.50, *z* = 0.464, *P* = 0.643), modularity (*U* = 475.50, *z* =  − 0.496, *P* = 0.620), or the proportion of family members in their support network (*U* = 504.00, *z* =  − 0.108, *P* = 0.914).

**Table 2:** Network and interaction characteristics by group

Role	Network variable (median, SD)			
	Network size	Density	Modularity	Proportion family
All	9.00 (1.99)	0.75 (0.17)	0.03 (0.07)	0.61 (0.24)
Survivor	9.00 (2.33)	0.73 (0.19)	0.04 (0.78)	0.61 (0.21)
Care partner	8.00 (1.51)	0.77 (0.15)	0.03 (0.06)	0.65 (0.27)
Total number of interactions by support type				
	Emotional	Informational	Belonging	Instrumental
All	1079	691	1284	948
Survivor	633	412	708	535
Care partner	441	272	568	409
NA*	5	7	8	4
Number of network members expected to provide each support type (baseline)				
	Emotional	Informational	Belonging	Instrumental
All	439	389	361	270
Survivor	194	168	145	123
Care partner	211	191	190	121
Number of interactions in each concordance category				
	Expected/received	Expected/not received	Not expected/received	Not expected/not received
All	766	699	206	444
Survivor	327	303	128	226
Care partner	398	321	72	192
NA*	41	75	6	26

*Participants who did not identify members who provided the specific type of support

Survivors identified 630 unique support network members who provided at least one of the four support types measured, and care partners identified 713 unique members. Both survivors and care partners reported having more members providing emotional support compared with other forms (30.79% of survivors’ members vs. 29.59% of care partners members), followed by informational (26.67% vs. 26.79%), belonging (23.02% vs. 26.65%), and instrumental (19.52% vs. 16.97%).

### Support interaction characteristics

Participants reported a total of 4002 interactions with network members over the 21 days of daily reporting (median, 53.5; SD, 27.16; range, 15–144); survivors reported a total of 2288 support interactions (median, 59.5; SD, 29.39; range, 21–122) and care partners reported 1690 (median, 51; SS, 27.28; range 15–144); this did not differ across groups (*U* = 458.00, *z* =  − 0.73, *P* = 0.468). On average, participants reported not interacting with 17% of their baseline-identified support network members during the study (median, 14.29%; SD, 1.684). Of the members with whom they interacted, 32.7% of support interactions were reported as not helpful (median, 28.6%; SD, 0.214; range 0–75). There were no significant differences between survivors and care partners on either the proportion of network members with whom they did not interact (*U* = 397.50, *z* =  − 1.57, *P* = 0.116) or unhelpfulness (*U* = 573.00, *z* = 0.82, *P* = 0.411).

### Concordance of expected vs. reported support

**Fig.1:**
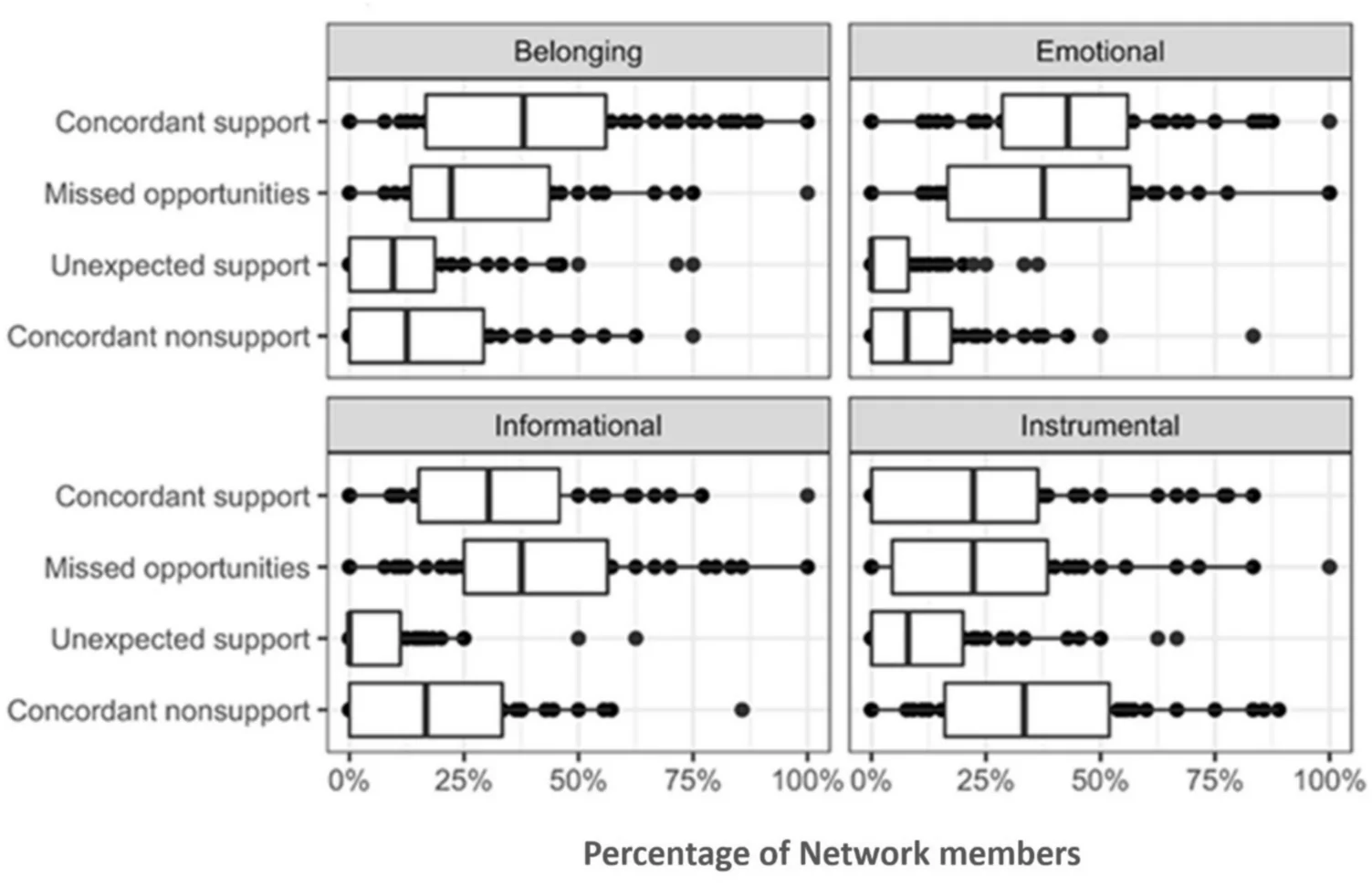
Concordance of expected vs. received support by type

Figure 1 presents the overall percentage of concordant network members for each support category. Overall, across all support types, participants’ baseline expectations matched future interactions and perceived outcomes 38% of the time. Emotional support had the highest median proportion of concordant support (median, 43%; IQR = 29–56), and instrumental support had the highest proportion of concordant nonsupport (median, 33%; IQR, 16–53). Informational (median, 38%; IQR, 25–56) and emotional support (Median, 38%; IQR, 17–56) had the most missed opportunities. Belonging support had the highest proportion of unexpected support (median, 10%; IQR, 0–19).

#### Concordance scores

Survivors had a mean concordance score of 2.29 out of a possible 4.0, based on the proportion of their network members for whom their daily prospective interaction reports matched their baseline expectations of receiving or not receiving each specific type of support from that member (SD = 0.631; range, 0.43–3.14). Caregivers’ mean concordance score was 2.68 (SD, 1.86; range, 1.83–4.0; Table 3). Survivors and care partners also did not differ significantly in their concordance scores (*U* = 502.5, *z* =  − 0.13, *P* = 0.9), nor were there significant differences based on participant gender (*U* = 467.5, *z* =  − 0.01, *P* = 0.99) or age (*U* = 448.50, *z* = 0.85, *P* = 0.397).

**Table 3:** Participant concordance scores by role, gender, and age

Concordance by role			
Role	Mean	Median	SD
All	2.27	2.27	0.58
Survivor	2.25	2.32	0.66
Care partner	2.39	2.32	0.50
Concordance by gender			
Gender	Mean	Median	SD
Men	2.44	2.36	0.41
Women	2.25	2.23	0.68
Nonbinary	1.83	1.83	NA
Concordance by age			
Age	Mean	Median	SD
< 40	2.37	2.25	0.53
40 +	2.27	2.41	0.64

#### Association of concordance scores with demographic, network, and interaction characteristics

Table 4 shows the results of our correlation analyses. Participants’ concordance scores were not correlated with age, income, or education. Similarly, concordance scores were not associated with the size, density, or modularity of survivors’ or care partners’ networks or the number of family members in the network.

**Table 4:** Correlations between concordance scores and demographic, network, and interaction variables

Variables		Spearman’s rho	*P* value	95% CI (lower)	95% CI (upper)
Age		–0.08	0.53	− 0.33	0.18
Education		0.15	0.24	− 0.09	0.39
Income		0.08	0.52	− 0.18	0.33
Network size		–0.01	0.92	− 0.28	0.23
Density		0.003	0.98	− 0.25	0.27
Modularity		–0.03	0.79	− 0.31	0.22
Proportion of family		0.03	0.82	− 0.25	0.32
Total interactions		0.57	0.001*	0.36	0.73
Non-interacting members		–0.41	0.001*	− 0.62	− 0.17
Unhelpful interactions		–0.59	0.001*	− 0.75	− 0.39
Total interactions	Unhelpful interactions	–0.47	0.001*	− 0.64	–0.24
Non-interacting members	Unhelpful interactions	0.64	0.001*	0.46	0.77

*Significance set at < 0.05

Participant concordance was significantly and positively correlated with the total number of interactions participants reported across the 21 days of daily diary reports (*r* = 0.57, *P* < 0.001). In contrast, concordance was negatively correlated with both the proportion of network members identified at baseline as providing support but with whom they did not interact over the 3-month study span (*r*_s_ =  − 0.41, *P* < 0.001) and with the proportion of reportedly unhelpful interactions (*r*_s_ =  − 0.59, P < 0.001). Further, the total number of interactions was negatively correlated with unhelpful interactions (*r*_s_ =  − 0.47, *P* < 0.001), while the proportion of non-interacting members was also positively correlated with unhelpful interactions (*r*_s_ = 0.64, *P* < 0.001), suggesting that those participants with more interactions experienced a lower proportion of unhelpful interactions. In contrast, those with more non-interacting members tended to report more unhelpful interactions.

## Discussion

In summary, our sample of cancer survivors and care partners revealed limited concordance between participants’ baseline perceptions of which network members typically provided specific types of support and the support they received in interactions with network members over time. Across all participants and support types, only 38% of reported interactions aligned with participants’ baseline perceptions. When concordance was calculated at the individual level, participants’ average score was 2.3, or 57%, reflecting the proportion of support interactions that matched their perceptions across the four support types. Although participants considered routine sources of support, they did not interact with 17% of these support network members over the study period, and 33% of their reported interactions were perceived as unhelpful. This suggests a gap that may reflect several factors relevant to cancer survivorship and caregiving. First, support needs and the availability of network members can shift over time, so baseline perceptions may not accurately track evolving demands [24, 25]. Second, some network members may have been willing but were never approached, or the survivor–care-partner dyad did not require certain kinds of support during the study period. Also, activating help can be complicated by distance, daily treatment schedules, role changes, or reluctance to seek assistance [26, 27]. Together, these dynamics—changing needs, selective engagement of help, and evolving interaction patterns—shape the observed concordance between baseline perceptions and reported support interactions [28].

Interestingly, our results also suggest that participants with fewer interactions with social support network members may be more likely to experience a mismatch between their perceptions of available support and the support they received. This aligns with social network theory, which posits that larger, denser, more interconnected networks, especially those that include a mix of family and non-family members, can provide greater access to support [29]. In our study, however, we did not find significant associations between structural network characteristics (size, density, modularity, or proportion of family members) and the concordance between participants’ baseline perceptions and their reported support interactions. These findings suggest that, for cancer survivors and care partners, structural aspects of support networks may not fully explain how support is experienced. For example, a large and tightly connected network may still fail to provide useful support if key members are unavailable, unwilling, or unclear about the survivor’s or care partner’s needs [30, 31]. This highlights the importance of looking beyond structural features to assess how support is mobilized and perceived in the everyday context of survivorship and caregiving over time, to better understand these dynamics.

We found no associations between concordance and demographic characteristics such as age, education, or income. While this may reflect characteristics of our sample, it also suggests that demographic factors alone may not fully explain how support is experienced in the context of cancer survivorship and caregiving. For example, two survivors with similar income and education levels may both identify a sibling as a key support person. Yet, one sibling may consistently provide transportation, attend appointments, and check in regularly, while the other may be emotionally supportive but unavailable due to distance or competing responsibilities. Despite similar demographic profiles and perceived support roles, their actual experiences of support could differ. This underscores the need to examine how support is engaged over time, not just who is expected to provide what type of support [32].

While we did not find associations between concordance and demographic or structural network characteristics, we did find significant relationships among variables reflecting participants’ perceptions of support over time, such as the total number of interactions and mismatches between expected and reported support. These findings highlight the importance of individuals’ perceptions in shaping how support is recognized and engaged [33, 34]. For example, a care partner who anticipates emotional support from a sibling may feel isolated if interactions remain logistical, or a survivor expecting practical help from a friend may not reach out again if their support is inconsistent. Approaches that assess perceptions across time and help survivors and care partners identify where expectations may align or misalign with actual support, or how specific events or circumstances may change routine dynamics, may be especially helpful in planning, addressing unmet needs, and strengthening support engagement. Through actual engagement with their social support networks, survivors and care partners learn whether the support they expect is available, revealing gaps or alignment between their perceptions and expectations, their needs, and the responses of others [15, 35].

Although most participants identified multiple sources of support and received help across domains, they were more accurate in anticipating emotional and instrumental support than informational or belonging support. This may reflect how some support needs are more visible, seem more urgent, or are more easily conveyed by survivors and care partners to social support network members. Emotional support can often be provided from a distance, such as check-ins, expressions of empathy, or verbal encouragement, while instrumental support tends to require physical presence and planning. Previous research on social support needs and perceived support among cancer survivors and cancer care partners frequently finds that emotional and instrumental support are perceived as most needed, helpful, and accessible, particularly during the acute survivorship phase while individuals undergo active treatment. Family members and supporters adjust to more intensive care partner roles [36, 37].

Informational or belonging support, in contrast, may be harder to anticipate, more difficult to seek or provide, or less consistently enacted. Members of the support networks of cancer survivors and care partners—especially if they have not experienced cancer themselves—may feel unsure about how to offer different kinds of support, may be reluctant to create a perceived burden by asking, and may offer what seems more comfortable or familiar [38]. Survivors and care partners, in turn, may be reluctant to ask for what they need, or to tell family or friends that the support offered is not helpful [26].

Support network members may feel more confident in providing emotional or instrumental support; however, instrumental and belonging support require knowledge and experience that not all network members can provide, or specific types of relationships, or support attempts may be perceived as less helpful. The life course of individual cancer survivors is also likely to be a factor. For example, young adult cancer survivors progressing through developmental milestones may seek to distance themselves from close family members or friends who they feel see them through a “cancer lens.” They may gain a greater sense of belonging and normalization through their connections with extended social and friend networks comprised of people who are not as focused on their health or cancer history. If their social support networks are comprised mainly or only of family connections, forming connections with non-family members can be more challenging, requiring more effort and resources, especially if their survivorship experience includes disruptions in conventional social or cultural milestones, such as college, work, or dating [26].

Moreover, more peripheral or occasional relationships—what social network theory defines as “weaker ties” compared with close family or friends—are often important sources of informational and belonging support; this can include health care providers, social or sport club members, connections through religious or spiritual practice, or affiliates encountered in routine recreational or social activities. Social selectivity theory (SST) posits that older adults actively “prune” their social networks to focus on their emotional support needs, leading to more restricted networks that lack diversity in connections and support resources, and negatively impact social activity and physical health. [39, 40] Experiences of both cancer and caregiving can also have profound effects on relationships and social connections, leading to progressive dwindling of social networks, constriction of support resources accessed through social connections, and social isolation [41, 42]. Helping older cancer survivors maintain or strengthen more diverse social support networks may be protective against functional impairment and help maintain well-being [43]. This is where formal or newer relationships and connections, established in the context of cancer survivorship rather than before, may be helpful, i.e., support groups, navigator programs, effective communication with and by providers.

These distinctions highlight the limitations of static assessments based solely on demographic characteristics or structural features, such as network size or density, or measures of social support that do not account for the more complex dynamics of how social support networks evolve [6]. Survivors and care partners may have similarly sized networks yet experience them very differently based on how needs change and how support is enacted over time. This can make assessing the sources and availability of different types of support resources over time challenging for survivors, their care partners, and healthcare providers. Approaches that incorporate time-based, perception-informed assessments—such as mapping perceived support roles, tracking how needs are met or missed across time points, or reflecting on where expectations diverge from lived experience—may offer greater insight into support dynamics than approaches that categorize networks by composition alone [36]. Both cancer survivors and care partners report positive and negative shifts in their social support networks [42]. Helping survivors and their care partners develop a better understanding of how their individual social support networks function, how they intersect, and potential gaps and areas of strength could reduce social isolation and functional impacts on physical and mental health by helping them navigate negative changes and build on positive ones.

### Limitations

We found no association between structural network characteristics and concordance scores. One explanation is that our ability to detect structural effects was limited by characteristics of the sample, including potentially constrained variability in network size, density, or composition. It is also possible that structural features alone do not fully explain how support is experienced in the context of cancer survivorship and caregiving. However, the observed associations with functional aspects of support—such as total interactions and reports of unhelpful or absent support—suggest that low concordance reflects meaningful differences in how support was engaged and perceived, beyond what structural similarity alone can account for.

Additionally, our study design may not have captured the extent to which social support network interactions may vary over time. Although we assessed interactions over 21 days across three months, we may still have missed essential interactions. We also did not limit the stage of cancer survivorship (i.e., near diagnosis or engaging in new treatment decisions), which may have impacted the specific support needs of our sample. More detailed information about the context of interactions or insights from network members could provide richer information about failed support attempts or why interactions were not perceived as supportive. Finally, we assessed support as perceived by participants, rather than the attempted support from network members. Research suggests that network members may have felt that they were offering effective support, but participants did not necessarily notice or perceive this in the same way [44, 45].

## Conclusion

Our approach and findings highlight the potential of knowledge gained by moving beyond retrospective, structure-focused, or availability-based assessments of social support, which often assume that identified social support network members will fulfill expected roles and provide expected types of support when needed. Even when survivors and caregivers identified available sources of support, their expectations often did not align with the actual experience of support over time. This underscores the value of approaches that attend to the dynamic and relational aspects of support—how it is perceived, engaged, and changes throughout the cancer experience and life course. Interventions that help survivors and care partners reflect on, recalibrate, and communicate their support needs may improve alignment between anticipated and actual support. In the context of cancer care, such strategies could enhance the effectiveness of informal support networks, reduce unmet needs, promote engagement with formal support resources when needed, and improve quality of life during active treatment and beyond.

## Data Availability

The dataset analyzed during this report are available from the corresponding author upon reasonable request.
